# Progress on the Fabrication of Superconducting Wires and Tapes via Hot Isostatic Pressing

**DOI:** 10.3390/ma16051786

**Published:** 2023-02-22

**Authors:** Zhenyu Lei, Chao Yao, Wenwen Guo, Dongliang Wang, Yanwei Ma

**Affiliations:** 1 Key Laboratory of Applied Superconductivity, Institute of Electrical Engineering, Chinese Academy of Sciences, Beijing 100190, China; 2 University of Chinese Academy of Sciences, Beijing 100049, China; 3 Institute of Electrical Engineering and Advanced Electromagnetic Drive Technology, Qilu Zhongke, Jinan 250013, China

**Keywords:** hot isostatic pressing, overpressure, BSCCO, MgB_2_, iron-based superconducting wires

## Abstract

Fabrication of high-performance superconducting wires and tapes is essential for large-scale applications of superconducting materials. The powder-in-tube (PIT) method involves a series of cold processes and heat treatments and has been widely used for fabricating BSCCO, MgB_2_, and iron-based superconducting wires. The densification of the superconducting core is limited by traditional heat treatment under atmospheric pressure. The low density of the superconducting core and a large number of pores and cracks are the main factors limiting the current-carrying performance of PIT wires. Therefore, to improve the transport critical current density of the wires, it is essential to densify the superconducting core and eliminate pores and cracks to enhance grain connectivity. Hot isostatic pressing (HIP) sintering was employed to improve the mass density of superconducting wires and tapes. In this paper, we review the development and application of the HIP process in the manufacturing of BSCCO, MgB_2_, and iron-based superconducting wires and tapes. The development of HIP parameters and the performance of different wires and tapes are reviewed. Finally, we discuss the advantages and prospects of the HIP process for the fabrication of superconducting wires and tapes.

## 1. Introduction

### 1.1. Development of Superconducting Wires

Superconductors were first discovered over 100 years ago, and thousands of different superconductors have since been found. Considering current-carrying performance in the magnetic field, superconducting transition temperature, mechanical performance, and fabrication difficulty, only a few superconducting materials have practical value, such as NbTi, Nb_3_Sn, Bi_2_Sr_2_Ca_2_Cu_3_O_10_ (Bi-2223), Bi_2_Sr_2_Ca_1_Cu_2_O_8_ (Bi-2212), REBa_2_Ca_2_O_7-δ_ (REBCO, RE = Y, Gd, Dy, Eu), MgB_2_, and iron-based superconducting materials [1].

In order to use superconducting materials in practical applications, it should be processed into wires or tapes first. The practical superconducting materials can be divided into alloy superconductors (such as NbTi), intermetallic compounds (such as Nb_3_Sn, Nb_3_Al, and MgB_2_), and ceramic superconductors (such as copper-based oxides and iron-based pnictides and chalcogenides) according to their mechanical properties. Alloy superconductors have good plasticity and are easily deformed, while intermetallic and ceramic superconductors have high hardness and brittleness and are difficult to mold through plastic deformation. Therefore, the powder-in-tube (PIT) method or coated conductor technology is usually used [2].

The PIT method is the most widely used preparation method for intermetallic and ceramic superconducting wires, including the preparation of superconducting precursor powder, the packing of the precursor into a metallic tube, cold machining, and heat treatment. This method was initially applied to the fabrication of YBCO superconducting wires [3], but it was replaced by other methods. The PIT method has been successfully used in the commercial preparation of Bi-based cuprate (BSCCO) superconducting wire and MgB_2_ superconducting wire due to its simple process and low cost [2,4]. The PIT method is also widely used in the fabrication of new iron-based superconducting wires.

In the PIT method, a series of machining processes and heat treatments is applied to the superconducting wires with the aim of densifying the superconducting core, improving the grain alignment, and promoting phase reaction. However, densification of the superconducting core is limited in the traditional machining process. The low density of the superconducting core and various defects (such as pores and cracks in the superconducting core) lead to poor grain connectivity, which seriously degrades the current-carrying performance of superconducting wires. Therefore, some new high-pressure processes have been investigated in the fabrication of superconducting wires for further enhancements to mass density, including hot pressing (HP), cold isostatic pressing (CIP), and hot isostatic pressing (HIP) sintering. However, HP is not suitable for the fabrication of long or round-shaped wires. On the other hand, as a cold processing technique, CIP is less effective at eliminating the residual pores and cracks in the superconducting core compared with HIP. Therefore, HIP is a promising option for improving the critical current density (*J*_c_) of superconducting wires and tapes.

### 1.2. Development and Application of the Hot Isostatic Pressing Process

**Figure 1:**
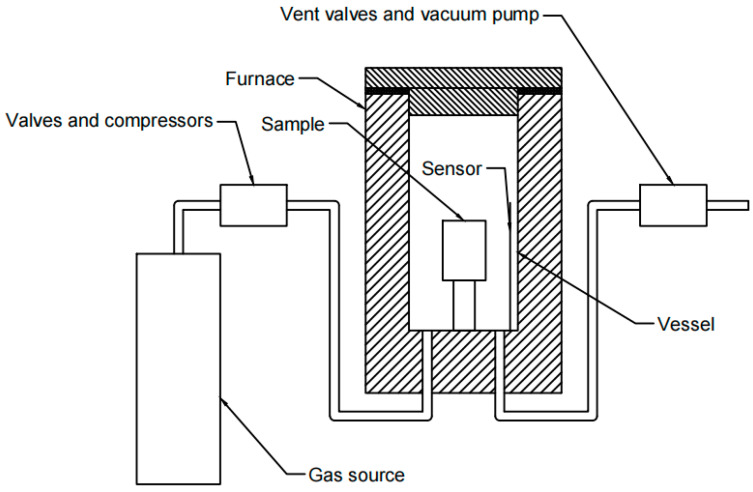
Schematic diagram showing the main structure of an HIP system.

Hot isostatic pressing (HIP) is a kind of isostatic pressing process in which liquid or gas is used as a pressure transmission medium to press the workpiece in order to reduce porosity and improve mass density. The pressure applied to each position on the surface of the workpiece is equal, thus it is called isostatic pressing. According to different temperatures, isostatic pressing can be divided into three approaches: cold isostatic pressing, warm isostatic pressing, and HIP. The temperature of HIP is usually in the hundreds to thousands of degrees Celsius. Additionally, the pressure of HIP can be up to hundreds of MPa, or even several GPa. Based on different process requirements, the HIP process can use different gases as pressure media, such as argon, helium, nitrogen, and oxygen. An HIP device generally consists of a furnace body, vacuum system, gas source and gas compression system, exhaust system, and electrical control system. The main structure of an HIP device is shown in Figure 1. In the process, the furnace body is provided with high pressure gas through the gas source and gas compression system. The pressure in the furnace is monitored by the pressure control system, and the intake and exhaust systems are adjusted to stabilize the pressure in the furnace body. The temperature control system is used to heat the furnace body and stabilize the temperature in the furnace in order to provide high temperature and a high-pressure heat treatment environment for the workpieces [5].

The most representative application of HIP is powder densification. The powders are sealed in a metal, glass, or ceramic sheath and then heat-treated under high temperature and pressure. The high pressure is transmitted through the sheath, which is softened by high temperature, to the powder inside. This can further improve the density of powder, promote powder particle contact, restrain the thermal expansion of the gas in the gap at the same time, and provide a favorable environment for element diffusion and recrystallization. However, if the sheath is not entirely sealed, that high-pressure gas enters the sheath, the pressure difference will disappear, and the purpose of densification and sintering will not be achieved. To transfer the pressure to the powder inside the sheath, it is necessary to ensure that the pressure is greater than the yield strength of the sheath material at the set high temperature. Therefore, the selection of an appropriate coating material is critical. In addition, there is also an HIP process without sheaths, whose essence is the self-sealing effect of the closed-cell state formed on the surface of the material after initial sintering. It is often used for the preparation of workpieces with complex shapes. The shape and particle size of the powder affect filling density, and the available particle size should be less than 500 μm. At the same time, the process of filling the powder is supplemented by vibration to improve the density and uniformity of the powder.

The HIP process was first invented by the Battelle Institute for the diffusion bonding of nuclear fuel components. With the help of high temperature and isostatic pressure, this process can achieve the good adhesion of many complex materials. Nowadays, HIP has been extended to many applications, including powder metallurgy, casting processes, ceramic material preparation, cermet composite preparation, and near-net-shape forming [6,7]. For these manufacturing fields, the advantages of HIP are unique. In powder metallurgy and casting treatment, it helps to achieve a density that is close to the theoretical density and contributes to fine grain structure, thus improving fatigue and creep properties, ductility, impact strength, and uniformity. In addition, workpieces prepared by HIP powder metallurgy do not have the texture characteristics of forgings, so metallic materials that are difficult or impossible to forge or cast can be manufactured by using HIP. In the field of preparation of high-performance ceramic materials, HIP can densify powders that are difficult to densify under other processes and promote heterogeneous bonding at the same time. The HIP process also shows high precision in the control of workpiece shapes. Based on this characteristic, the near-net-shape forming process is developed to produce workpieces of various shapes and sizes, especially workpieces with complex external geometry or internal cavities, thus saving materials and reducing costs.

In the field of superconducting materials, the HIP process has been widely applied. In the early research of YBCO and BSCCO superconducting wires, the HIP process was applied to densify the superconducting filaments made using the PIT method [8,9,10]. However, a more recent approach has replaced the PIT method in the fabrication of YBCO superconducting wires, and the overpressure method was developed for the fabrication of BSCCO superconducting wires.

**Figure 2:**
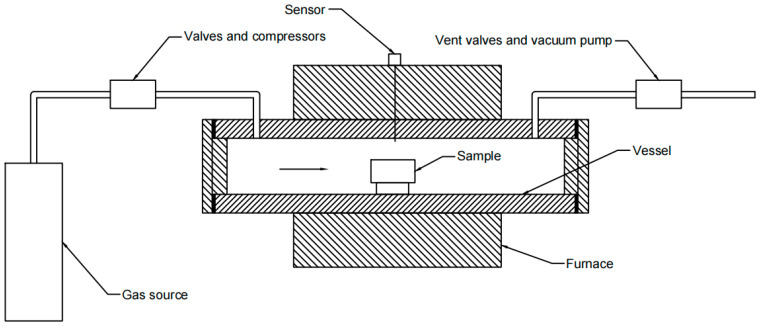
Schematic diagram showing the main structure of a flow overpressure system.

The overpressure method is a variant of HIP. Compared to HIP, the overpressure method reduces maximum pressure and maintains oxygen partial pressure (*p*O_2_) because the Bi-2223 phase will decompose in a pure argon atmosphere. Overpressure systems can be classified into two categories: static and flow strategies. The structure of a static system is similar to the HIP system. In the static system, the argon and oxygen are filled at the start of the heat treatment. The gas remains static during the heat treatment, so *p*O_2_ decreases because of the reaction between oxygen and the BSCCO filaments. The main structure of a flow system overpressure device is shown in Figure 2. In the overpressure process of BSCCO wires, Ar and O_2_ are pumped into the vessel and flow through the sample. The pressure of both Ar and O_2_ is strictly maintained by the flow system, so it is more suitable for the overpressure method [11].

For the superconducting PbMo_6_S_8_ wires, it was proven that the HIP of PbMo_6_S_8_ wires at 110 MPa increases transport *J*_c_, and the superconducting core can be consolidated with little grain growth [12,13]. At present, the HIP process and the overpressure method have been used in the fabrication of BSCCO, MgB_2_, and iron-based superconducting wires.

This article reviews the development and application of the HIP process in the fabrication of superconducting wires, including BSCCO, MgB_2_, and iron-based superconducting wires.

## 2. HIP Process in Superconducting Wires and Tapes

### 2.1. HIP Process in Superconducting BiSrCaCuO Wires and Tapes

BiSrCaCuO (BSCCO) superconductors were discovered in 1988 [14]. They are a series of superconductors that includes the high-temperature superconductors (HTSs) Bi_2_Sr_2_Ca_2_Cu_3_O_6+δ_ (Bi-2223) and Bi_2_Sr_2_CaCu_2_O_8+δ_ (Bi-2212), which are still used at present and have been industrialized. Critical current density (*J*_c_) is the main parameter of the performance of superconducting wires and tapes. The *J*_c_ of a BSCCO wire is significantly affected by the angle of grain boundaries and grain connectivity.

The forming of the superconducting core is a multi-complex and multi-process process with large deformation. Its purpose is to obtain high powder density, strong *c*-axis texture, and good forming uniformity. The powder-in-tube (PIT) method is the main method used in the fabrication of BSCCO wires [15]. It is mainly divided into three parts: precursor powder synthesis, mechanical deformation, and heat treatment. Precursor powder synthesis determines the ingredient of the superconducting core. Mechanical deformation improves grain connectivity by densifying the superconducting core and obtains a strong *c*-axis texture that decreases the angle of grain boundaries. However, in the final heat treatment process, a superconducting core that has been densified in the previous mechanical process may become less dense as the heat treatment inevitably creates cracks and voids in the superconducting core, reducing transport *J*_c_. Kametani et al. demonstrated that the porosity of Bi-2212 filaments is caused by bubbles aggregated with gas remaining in the precursor powders [16]. These bubbles block the connection of superconducting filaments, reducing 20–30% of critical current (*I*_c_) in 1 m samples compared to 5–10 cm samples.

**Figure 3:**
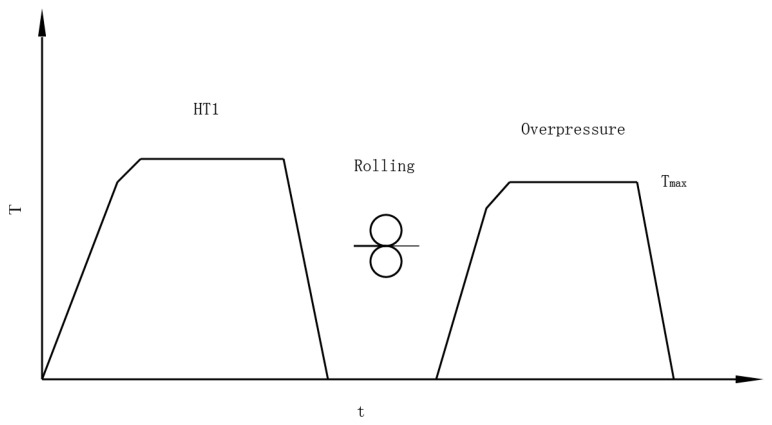
Heat treatment schedule for Bi-2223 wires. The Bi-2223 wires are rolled after the first heat treatment and then treated using the overpressure method.

While regular heat treatments fail to realize both a high-purity superconducting phase and high-density superconducting core, a variant of HIP called the overpressure method has been developed to solve this problem. A regular heat treatment schedule for Bi-2223 wires is shown in Figure 3.

The overpressure method was first reported for the treatment of Bi-2223 by Rikel et al. in 2001 [17]. They applied overpressure on Ag-sheathed Bi-2223 tapes under a pressure of 17.5 MPa for 36 h at 815 °C, achieving *I*_c_ of 100 A (77 K, 0 T).

In 2005, Kobayashi et al. from Sumitomo Electric Industries realized the commercial production of Bi-2223 multi-core superconducting tapes through the controlled overpressure (CT-OP) method [18,19]. Silver alloy-sheathed Bi-2223 wires were prepared with the precursor powder of Bi-2212 using the PIT method. After the first heat treatment and rolling, the Bi-2223 wires were densified by CT-OP under a pressure of 0.1–30 MPa with *p*O_2_ of 4–20 kPa. The experimental results show that the *I*_c_ of the 1500 m wire after CT-OP sintering reaches 100 A (77 K, 0 T), while the *I*_c_ of atmospheric-pressure sintering is 75 A.

**Figure 4:**
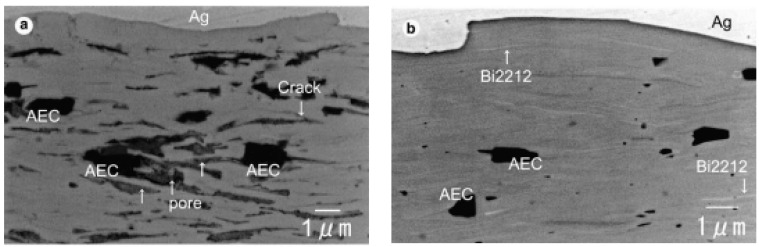
Comparison of SEM secondary electron micrographs of (**a**) Bi-2223 normal-pressure filament and (**b**) overpressure filament. AEC is alkaline earth cuprate, such as (Ca,Sr)-Cu-O [19]. Adapted with permission from Ref. [19]. 2005, Elsevier.

The improvements in the transport *J*_c_ performance of Bi-2223 wires sintered by the CT-OP method are associated with increased core density, reduced secondary phases, and the recovery of cracks and pores, as is shown in Figure 4. It also improved the mechanical performance of the Bi-2223 wires. The critical tensile stress of Bi-2223 wire is enhanced from 85 MPa to 154 MPa after CT-OP sintering.

In 2007, the *I*_c_ performance of a 700 m long wire prepared using their improved process reached 197 A (77 K, 0 T), and the engineering *J*_c_ reached 2.1 × 10^4^ A cm^−2^ [20]. At present, Bi-2223 wires produced by CT-OP with *I*_c_ of 200 A have been mass produced and widely applied [21].

Tajima et al. studied the effects of initial sintering and HIP treatment under reducing *p*O_2_ on the transport *J*_c_ performance of Bi(Pb)2223 bulks and silver-sheathed multi-filament tapes [22]. Almost single-phase Bi(Pb)2223 containing small grains and impurities was obtained in the first sintering at 825 °C for 24 h in 2% O_2_. Overpressure treatment under a total pressure of 10 MPa and *p*O_2_ of 3 kPa showed the highest *I*_c_ for tapes (up to 124 A), while *p*O_2_ of 2 kPa led to large amounts of Bi(Pb)2212 remaining. It was suggested that sintering under moderately low *p*O_2_ is effective for Bi(Pb)2223 tapes, but the reduction of *c*-axis texture needs improvement.

The typical parameters of Bi-2223 tapes fabricated by the overpressure process are shown in Table 1 [17,18,22]. It is illustrated that the pressure of the overpressure process varies from 10 MPa to 30 MPa, which is lower than that of the normal HIP process. *p*O_2_ and temperature are restricted in a small range because the formation of the Bi-2223 phase is sensitively related to these parameters.

The overpressure method is also applied in the fabrication of Bi-2212 wires. In 1997, Reeves et al. used the overpressure method for the heat treatment of Bi-2212 wires [23,24]. The wires were heated at 895 °C under pressures of up to 0.9 MPa with *p*O_2_ = 0.1 MPa. The wires were then fast-cooled at a rate of 120 °C/h. The results showed that the amount of bubbles in the Ag sheath decreased with increasing pressure. These bubbles were caused by expansion of the gas in the superconducting filaments.

In 2011, Jiang et al. applied the CIP method to Bi-2212 wires, but the heat treatment was still atmospheric [25]. The heat treatment of Bi-2212 wires had been dominated by atmospheric sintering until 2014, when Larbalestier et al. fabricated Bi-2212 round wires using the overpressure method at 10 MPa [16,25,26,27]. *J*_e_ reached almost 1.0 × 10^3^ A mm^−2^ (4.2 K, 5 T). It was demonstrated that overpressure heat treatment improved the *J*_c_ of the Bi-2212 wires because of the densification of the superconducting filaments, grain alignment, and reductions in porosity. Miao et al. applied overpressure heat treatment combined with swaging and cold isostatic pressing to the fabrication of Bi-2212 wires [28]. A 1.2 m long sample was sintered under a pressure of 1 MPa. After overpressure treatment, the *J*_e_ of the sample increased from 470 A mm^−2^ to 550 A mm^−2^.

In 2019, Jiang et al. used overpressure heat treatment to produce Bi-2212 wires [29]. Two wires made with nGimat and MetaMateria powders were heated according to a schedule under 5 MPa total pressure with a *p*O_2_ of 0.1 MPa. *T*_max_ was varied from 884 to 894 °C to optimize the heat treatment. The wires made with nGimat powder achieved *J*_c_ of 6.64 × 10^3^ A mm^−2^ (4.2 K, 15 T) and *J*_e_ of 1.32 × 10^3^ A mm^−2^ (4.2 K, 15 T), which showed a 60% increase over Nexans powder.

**Figure 5:**
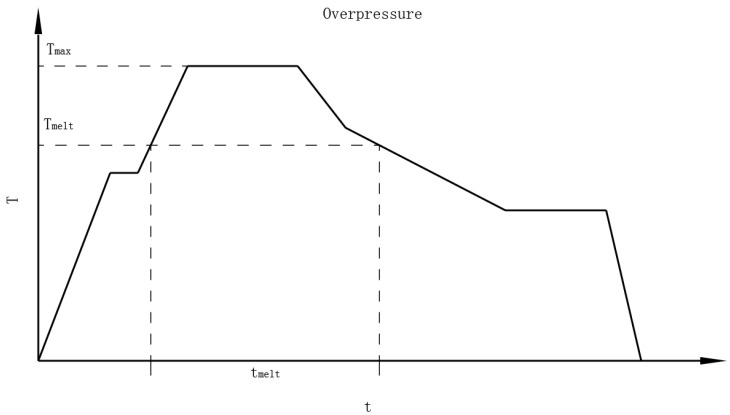
Heat treatment schedule for Bi-2212 wires. *T*_max_ is the maximum temperature and *T*_melt_ is the melting point of the Bi-2212 filaments. The time it takes for Bi-2212 filaments to melt is called *t*_melt_.

The maximum temperature of the overpressure heat treatment has a significant influence on the transmission performance of a Bi-2212 wire [30,31]. A regular heat treatment schedule for Bi-2212 wires is shown in Figure 5. In the overpressure treatment, the Bi-2212 filaments were in a powder state at first. When the temperature rises above the melting point, the Bi-2212 filaments melt into a liquid phase. The Bi-2212 filaments then cool down and form a solid phase. The time it takes for Bi-2212 filaments to melt is called *t*_melt_. The increase in *t*_melt_ will cause sausage filaments, accumulation of gas bubbles, and the growth of solid phases, while low *t*_melt_ will also decrease *J*_c_. *t*_melt_ has a direct correlation with *T*_max_. In Bi-2212 coil heat treatment, temperature gradients have been found across the coil. Therefore, an optimal *T*_max_ window needs to be found to optimize *J*_c_. Jiang et al. reported an optimal *T*_max_ window between 886 and 894 for an 85 × 18-filament Bi-2212 wire with a 1.2 mm diameter [31]. The optimal *T*_max_ window varies with the diameter of the wire, the quality of the precursor powder, and the size of the filaments. The cooling rate of the overpressure heat treatment also influences the crystallization process of the Bi-2212 phase. The *J*_c_ of Bi-2212 wires decreases with increasing cooling rate [30].

The parameters of Bi-2212 tapes fabricated using the overpressure process are shown in Table 2 [23,26,28,29,31,32]. It is shown that the pressure of the overpressure process of Bi-2212 wires varies from 1 MPa to 10 MPa, which is even lower than that of Bi-2223 wires. *p*O_2_ is a fixed value of 0.1 MPa, and optimal *T*_max_ is around 884~895 °C, which depends on the property of the wire. For future research, the relation between optimal pressure and the parameters of Bi-2212 wires should be further explored.

### 2.2. HIP Process in MgB_2_ Superconducting Wires

Superconductivity in MgB_2_ was discovered in 2001 [33]. MgB_2_ is a low-temperature superconductor, which has a critical temperature of 39 K and the advantages of low cost, low resistivity and specific gravity in a normal state, and low anisotropy [1]. MgB_2_ is a binary intermetallic compound with high hardness and brittleness, and its mechanical properties are similar to ceramics. The PIT method is the main method for preparing MgB_2_ superconducting wire. The improvement in the transport *J*_c_ performance of MgB_2_ mainly depends on improving the upper critical field, flux pinning, and grain connectivity. The HIP process can effectively improve the superconducting core’s density and grain connectivity, which is a significant development direction for improvements in the transport performance of MgB_2_.

The HIP process in the fabrication of MgB_2_ bulks has been reported since superconductivity in MgB_2_ was discovered. It was discovered that the HIP process leads to good grain connectivity and the high transport *J*_c_ performance of MgB_2_ bulks [34,35].

In 2003, Serquis et al. fabricated a single-filament MgB_2_ wire using the PIT method and the HIP process. The wire was sheathed in stainless steel with an external diameter of 0.8–1.4 mm. The wire was cut into 10 cm long samples, which were HIP processed at 900 °C under 200 MPa for 30 min. The HIP samples showed high magnetic *J*_c_. However, transport *J*_c_ was low in a magnetic field under 9 T. This may be due to the poor connectivity between the stainless-steel sheath and MgB_2_ core caused by tensile stresses [36,37,38,39].

The Gajda group of the Institute of High Pressure Physics at the Polish Academy of Sciences have conducted research on the influence of the HIP process on MgB_2_ properties. In 2015, MgB_2_ wires prepared at Hyper Tech Research, USA, with continuous tube forming and filling were processed through HIP by the Gajda group [40]. The samples were annealed at 700 °C for 15 min at a pressure of 1 GPa and 0.1 MPa, with one group of the samples undoped and the other group of the samples doped with 10% SiC.

**Figure 6:**
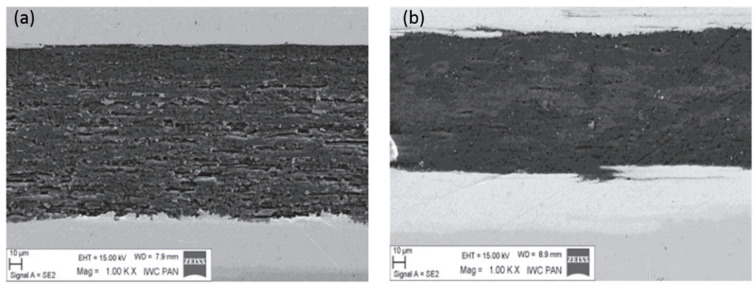
Comparison of SEM micrographs of (**a**) a normal-pressure filament and (**b**) an HIP filament [40]. Adapted with permission from Ref. [40]. 2014, IOP Publishing.

**Figure 7:**
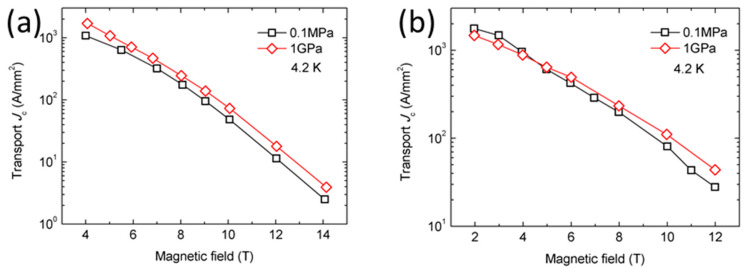
Transport *J*_c_ of the (**a**) undoped MgB_2_ samples at a pressure of 1 GPa and 0.1 MPa and (**b**) SiC-doped MgB_2_ samples at a pressure of 1 GPa and 0.1 MPa. Some data are based on [40].

The results of SEM showed that HIP increased the density and homogeneity of MgB_2_, and the high pressure significantly reduced the size of the voids, as is shown in Figure 6. Voids similar to coherence lengths in size are beneficial because they act as point-pinning centers to increase (pinning force) *F*_p_ and *J*_c_ in the intermediate magnetic field. The results in Figure 7 show that, for undoped samples, an HIP pressure of 1 GPa increases the high-field *J*_c_ of the sample by about 30% (7 T to 14 T) and the low-field *J*_c_ of the sample by about 36% (4 T to 7 T).

For samples doped with SiC, it can be seen in Figure 7 that HIP pressure of 1 GPa increased the high-field *J*_c_ of the sample by about 34% (7 T to 14 T) but decreased low-field *J*_c_ by about 16% (4 T to 7 T). High-pressure HIP increases *F*_p_ under a high magnetic field and reduces *F*_p_ under a low magnetic field. Kramer analysis shows that high pressure (1 GPa) increases the irreversible magnetic field (*B*_irr_) by about 1.2 T. The *B*_irr_ value of the 0.1 MPa sample is about 14.8 T, and the *B*_irr_ value of the 1 GPa sample is 16 T. The increase in the *B*_irr_ value in the sample obtained at high pressure may indicate that annealing under high Ar gas pressure affects C substitution for B and increases the density of dislocations.

**Figure 8:**
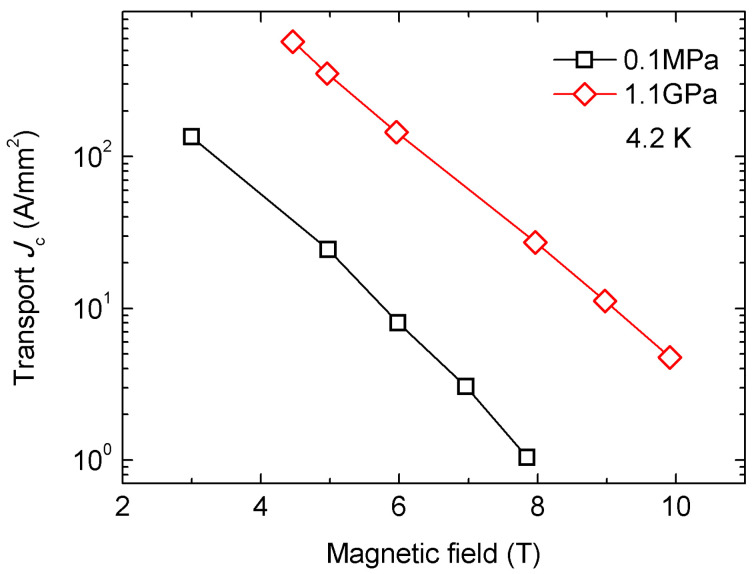
Transport *J*_c_ of the MgB_2_ samples at a pressure of 1.1 GPa and 0.1 MPa. Some data are based on [41].

In 2016, the Gajda group prepared a group of MgB_2_ samples with PVZ atomized magnesium and amorphous boron powder at the Institute of High Pressure Research in Warsaw [41]. The Mg and B were mixed with a stoichiometric ratio of 1:2 by rotary ball milling for 3 h under ambient conditions. The powder was pelleted with a mechanical press at 2 tons. Pellet size was close to 1–1.5 mm in height and the diameter was 8.8 mm. The pellets were filled in an iron tube with a diameter of 9 mm and an outer diameter of 12 mm. The unreacted MgB_2_ wire was then cold-drawn to a diameter of 0.9 mm (with a filling coefficient of approximately 40%). Unreacted MgB_2_ wires were HIP-treated at the Warsaw High Voltage Institute. One sample was annealed at 740 °C and 0.1 MPa for 40 min. The other sample was annealed at 740 °C for 40 min at a pressure of 1.1 GPa. HIP was conducted in a high-pressure pressure chamber under a 5N argon atmosphere. Figure 8 shows the transport *J*_c_ of the MgB_2_ samples. The results showed that annealing of MgB_2_ wires under high isostatic pressure (1.1 GPa) allows for more connections between grains, produces smaller MgB_2_ grains, and significantly eliminates the diffusion of iron atoms in the superconducting core. This process also improves grain connectivity and reduces the areas of unreacted Mg. The reduced amount of unreacted Mg indicates that the HIP process accelerates the formation of the MgB_2_ phase.

**Figure 9:**
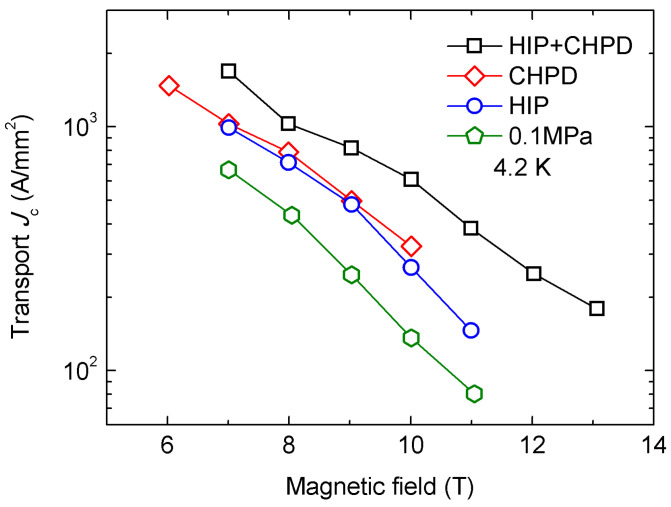
Transport *J*_c_ of the MgB_2_ samples heat-treated by HIP (1.4 GPa) + CHPD (1.8 GPa), CHPD (1.8 GPa), and HIP (1.4 GPa), as well as samples fabricated under 0.1 MPa. Some data are based on [42].

In 2017, Jie et al. demonstrated that cold high-pressure densification (CHPD) combined with HIP could more effectively increase mass density, reduce voids, and enhance *J*_c_ in both high and low magnetic fields [42]. The raw MgB_2_ wires were fabricated using the PIT method. The in situ powder was made with carbon-encapsulated amorphous boron powder and coarse magnesium powder. The powder was then filled into a Nb + Monel tube, and the tube was drawn to a wire with a 0.83 mm outer diameter. After drawing, the wires were pressed by a CHPD device at a pressure of 1.8 GPa from four directions at room temperature. The wires were then sintered in an HIP furnace under 1.4 GPa at 700 °C for 20 min. The medium of the HIP process was melted salt and BN instead of gas. Figure 9 shows the transport *J*_c_ of the MgB_2_ samples fabricated using different treatments. Consequently, the *J*_c_ performance of HIP + CHPD-treated wire was the best among all PIT MgB_2_ wires reported so far due to improved grain connectivity and enhanced flux pinning strength.

The parameters of MgB_2_ wires fabricated using the HIP process are shown in Table 3 (Parameters of MgB_2_ wires fabricated by the HIP process [40,41,42,43]). It is shown that the pressure of the HIP process for MgB_2_ wires exceeds 1 GPa because of the high hardness of MgB_2_ and the iron sheath. Relatively, the time required for the HIP process is reduced.

### 2.3. HIP Process in Iron-Based Superconducting Wires and Tapes

**Figure 10:**
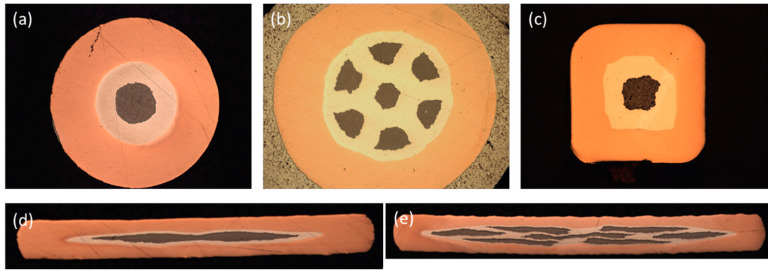
The cross sections of (Ba,K)Fe_2_As_2_ (**a**) round wire, (**b**) round seven-filament wire, (**c**) square wire, (**d**) tape, and (**e**) seven-filament tape.

Iron-based superconductors (IBSs) were first discovered by Hosono‘s research group at the Tokyo University of Technology in 2008, and the first IBS wire was successfully fabricated soon after [44,45,46,47]. Since then, research on the fabrication of IBS wires and tapes has been carried out by many research groups in China, the United States, Japan, and Europe. Iron-based superconductors can be broadly divided into several categories, including 1111-type, 122-type, 111-type, 11-type, and other new structural superconductors [48]. Superconducting wires and tapes with different shapes fit different applications. Figure 10 shows the cross sections of different forms of (Ba,K)Fe_2_As_2_ wires and tapes.

The PIT method is widely used in the fabrication of IBS wires. As well as other materials mentioned above, the HIP process can improve the density of the IBS core and reduce microcracks, thus significantly improving transport performance [49].

In 2012, Larbalestier et al. from Florida State University prepared a Cu/Ag composite-sheathed (Ba,K)Fe_2_As_2_ (Ba122) wire with high density and refined grains by combining cold isostatic pressing with HIP [50]. The *J*_c_ was about 10^4^ A cm^−2^ at 12 T. It was the first time HIP had been introduced in the preparation of IBS wire. Subsequently, they investigated the characteristics of HIP sintering of Ba122 at different temperatures [51]. One set of samples was sintered in an HIP furnace at 193 MPa pressure at 1120 °C for 12 h, before then being cooled to 900 °C at 4 °C/h for 20 h. Another group of samples was sintered for 20 h at 600 °C at 193 MPa in an HIP furnace and reground for 10 h at 600 °C at 193 MPa in an HIP furnace.

**Figure 11:**
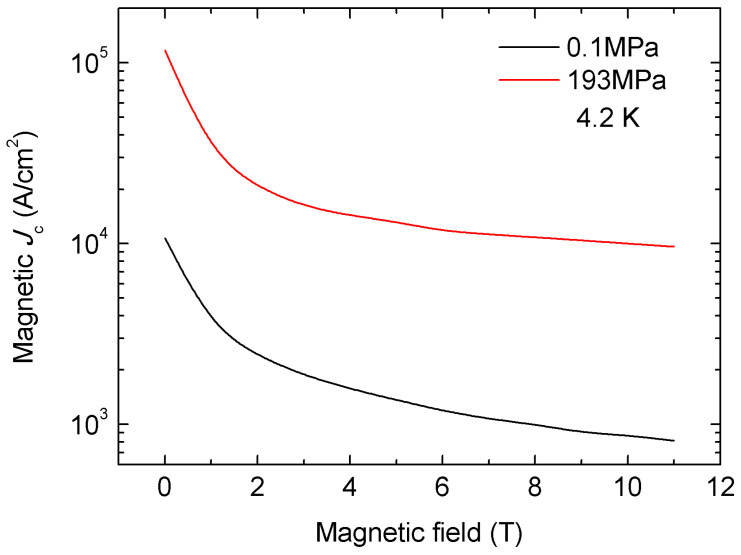
Magnetic *J*_c_ of the (Ba,K)Fe_2_As_2_ bulk samples heat-treated at 193 MPa and 0.1 MPa. Some data are based on [51].

The experimental results showed that the sample sintered at 1120 °C had a high density of 5.76 g cm^−3^, which is 98.5% of the theoretical density. At the same time, the sample had high phase purity, with impurity content of less than 1%. However, the sample did not exhibit transport performance because the crack and impurity phase separated to the grain boundary, thus blocking the intergranular current. The density of the sample sintered at 600 °C was 5.4 g cm^−3^, equivalent to 92% of the theoretical density, which is slightly lower than that of the sample sintered at 1120 °C; however, this was still significantly higher than the 4.0 g cm^−3^ of the sample sintered under atmospheric pressure. Figure 11 shows that magnetic *J*_c_ (4.2 K, 0 T) and *J*_c_ (4.2 K, 10 T) were up to 1.17 × 10^5^ A cm^−2^ and 8.9 × 10^3^ A cm^−2^_,_ respectively, which represents a ten-fold increase compared to the normal-pressure sintered samples. The magnetic *J*_c_ is calculated by magnetization measurements, while transport *J*_c_ is measured in a transport experiment. For wires and tapes, the transport *J*_c_ shows the practical transport performance. For bulks, the transport *J*_c_ is not available, so the magnetic *J*_c_ is tested to show local transmission performance. The results show that HIP can effectively improve the density of the superconducting filaments of Ba122 wire.

In 2014, Pyon et al. fabricated (Sr,K)Fe_2_As_2_ superconducting wires using an ex situ PIT method [52]. A mixture of starting materials with a ratio of Sr:K:FeAs = 0.6:0.44:4 was ground and sintered into precursor powder. The precursor powder was filled into a silver tube and drawn into 1.2 mm square wire. The silver wire was then put into a copper tube and redrawn into 1.2 mm square wire. Both ends of the wire were sealed in an arc furnace. The HIP method was the final heat treatment for the wires. They were heated at 700 °C for 4 h under a pressure of 120 MPa. The transport *J*_c_ of the (Sr,K)Fe_2_As_2_ HIP wire reached 1 × 10^5^ A cm^−2^ (4.2 K, 0 T) and 9.4 × 10^3^ A cm^−2^ (4.2 K, 10 T), which is almost 10 times larger than that of wires prepared under ambient pressure.

In 2016, Pyon et al. prepared 0.4 mm thick (Ba,K)Fe_2_As_2_ tapes by rolling 1.2 mm square wires, which are shown in Figure 10 [53]. The tape and the wire were processed using HIP under a pressure of 175 MPa at 700 °C and were held for 4 h. The transport *J*_c_ of the HIP wire reached 1.75 × 10^5^ A cm^−2^ (4.2 K, 0 T) and 2 × 10^4^ A cm^−2^ (4.2 K, 10 T), which is higher than previous works. The HIP tape showed a higher transport *J*_c_ of 2.54 × 10^5^ A cm^−2^ (4.2 K, 0 T) and 2.3 × 10^4^ A cm^−2^ (4.2 K, 10 T).

**Figure 12:**
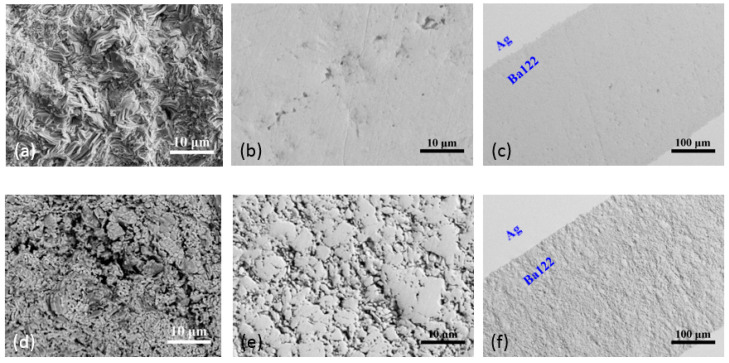
SEM and microscope images of (**a**–**c**) HIP-processed samples and (**d**–**f**) ambient pressure-sintered sample at different magnifications [56]. Adapted with permission from Ref. [56]. 2021, Shifa Liu.

**Figure 13:**
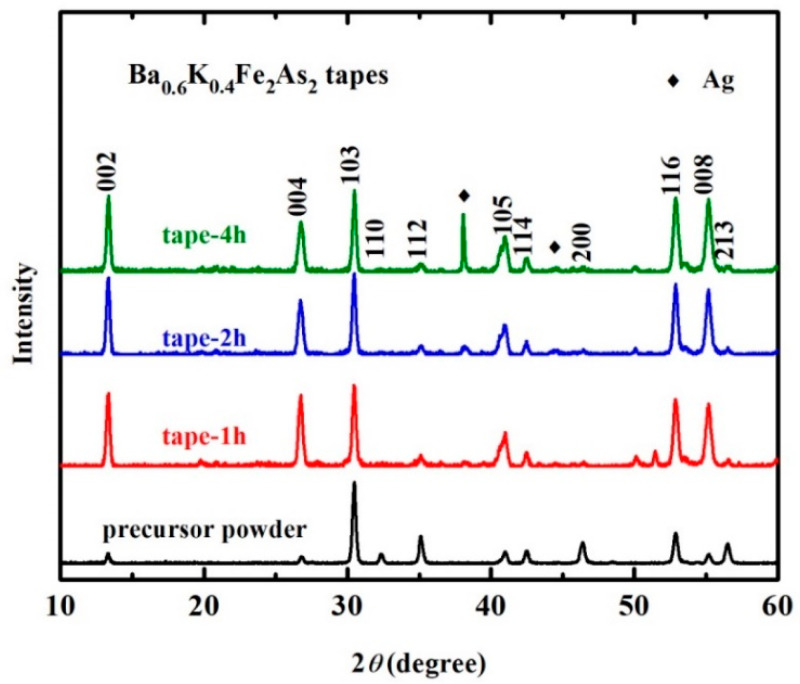
XRD patterns of precursor powder and the superconducting core of (Ba,K)Fe_2_As_2_ superconducting tapes with different sintering time [55]. Adapted with permission from Ref. [55]. 2019, IOP Publishing.

In 2017, Liu et al. fabricated (Ba,K)Fe_2_As_2_ superconducting wires using ex situ PIT and HIP methods [54]. The wires were sheathed in a Cu/Ag composite and drawn into round wires of 1.5 mm in diameter. The wires were then HIP-treated for 4 h at 700 °C under a pressure of 200 MPa. Figure 12 shows the highly dense structure in the (Ba,K)Fe_2_As_2_ cores of the HIP-processed sample compared with the ambient pressure-sintered sample. It is demonstrated that the HIP method can greatly reduce voids and cracks and improve grain connectivity. The density of the superconducting core *ρ* reaches 5.6 g cm^−3^, which is 96% of the theoretical density and much higher than 70~80% of ambient pressure-sintered samples. The transport *J*_c_ of the HIP wire reached 7.6 × 10^4^ A cm^−2^ (4.2 K, 0 T) and 9.4 × 10^3^ A cm^−2^ (4.2 K, 10 T). It is thought that the much lower degree of texture and non-uniform grain size are responsible for the much lower transport *J*_c_. An optimized HIP process was reported in 2019. The Cu/Ag-sheathed wires were flat-rolled into tapes 0.3 mm in thickness, which were then HIP-treated at 740 °C under a pressure of 200 MPa for 1, 2, and 4 h, respectively [55]. The highest transport *J*_c_ of the 1 h wire reached 1.9 × 10^5^ A cm^−2^ (4.2 K, 0 T) and 5.8 × 10^4^ A cm^−2^ (4.2 K, 10 T). The Vickers hardness value of the superconducting filaments reached 236 on average, which was considerably higher than the average of 138 for hot-pressed tapes, thus indicating good connectivity in a compact superconducting core. Suitable heat-treatment parameters guarantee a good recrystallization process, leading to good connectivity in the grains. Figure 13 shows the strong *c*-axis texture of the flat-rolled tape. The (00*l*) peak of the tape has stronger intensity than the precursor powder, indicating the *c*-axis orientation of the grains in the superconducting core. Considering the weak-link behavior in IBSs, the *c*-axis texture in the superconducting core may be one of the reasons for the high transport *J*_c_. It is also shown that a long sintering time may cause the diffusion of Ag into the superconducting core, thus reducing transport *J*_c_.

In 2020, Pyon et al. fabricated (Ba,Na)Fe_2_As_2_ superconducting round wires using PIT and HIP methods with similar process parameters to the HIP method used in their previous work [57]. The transport *J*_c_ of the HIP wires reached 2.04 × 10^5^ A cm^−2^ (4.2 K, 0 T) and 4.0 × 10^4^ A cm^−2^ (4.2 K, 10 T). The value under 10 T was higher than that of all IBS wires.

**Figure 14:**
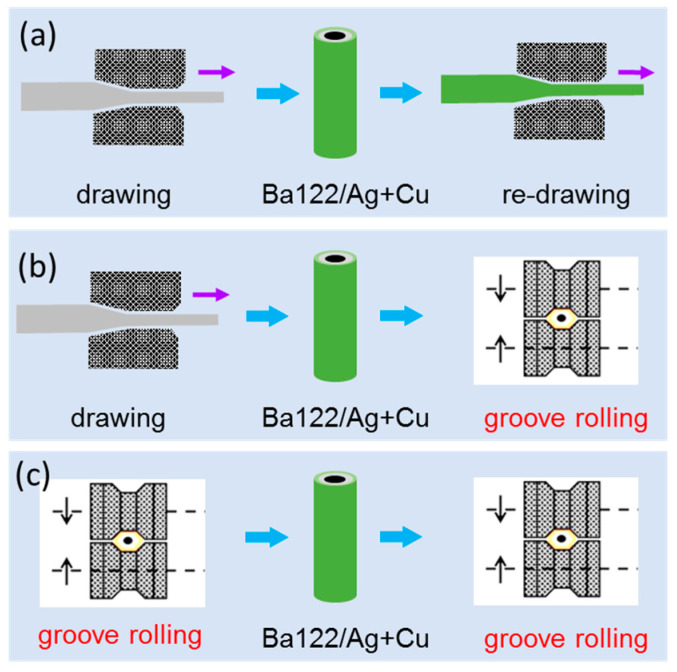
The introduction of groove rolling in (Ba,K)Fe_2_As_2_ wires. (**a**) Drawing is applied before and after a Ag wire is put into the Cu tube; (**b**) drawing and groove rolling are applied before and after a Ag wire is put into the Cu tube; (**c**) groove rolling is applied before and after a Ag wire is put into the Cu tube.

**Figure 15:**
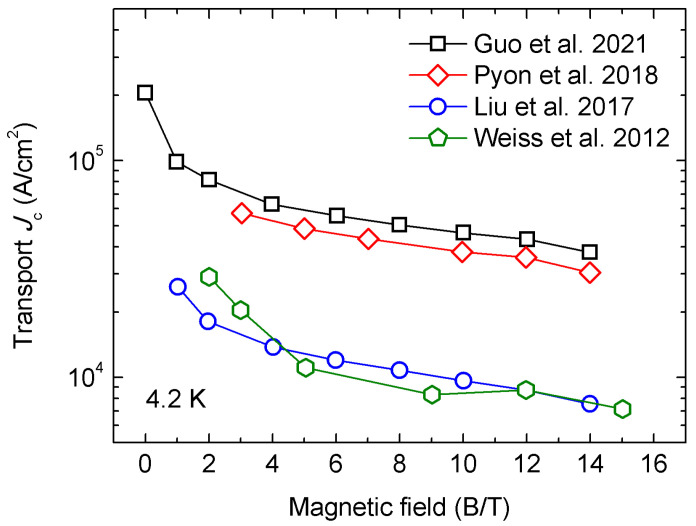
Comparison of the transport *J*_c_ performance of HIP (Ba,K)Fe_2_As_2_ superconducting wires from different groups [50,54,58,59].

In 2021, Guo et al. investigated the effect of groove rolling on the *J*_c_ performance of (Ba,K)Fe_2_As_2_ superconducting wires [58]. As is shown in Figure 14, three processes of the PIT method were tested in the experiment. The samples in which groove rolling was performed (both a Ba122/Ag tube and the composite Ba122/Ag/Cu tube) reached the highest transport *J*_c_ of 2.0 × 10^5^ A cm^−2^ (4.2 K, 0 T) and 4.7 × 10^4^ A cm^−2^ (4.2 K, 10 T), which was five times that of the drawing sample. Figure 15 shows that the groove-rolled wires reached higher transport *J*_c_ than previous ones. Higher density, better grain connectivity, and, especially, *c*-axis grain texture were introduced by the groove-rolling process, thus improving the transport *J*_c_ of the wires.

In the same year, Pyon et al. fabricated coils with round (Ba,Na)Fe_2_As_2_ and (Ba,K)Fe_2_As_2_ wires using the HIP method [60]. The (Ba,Na)Fe_2_As_2_ and (Ba,K)Fe_2_As_2_ coils were sintered under 200 MPa and 190 MPa, generating a magnetic field of 2.6 kOe and 2.5 kOe and achieving *I*_c_ of 60 and 66 A, respectively.

**Figure 16:**
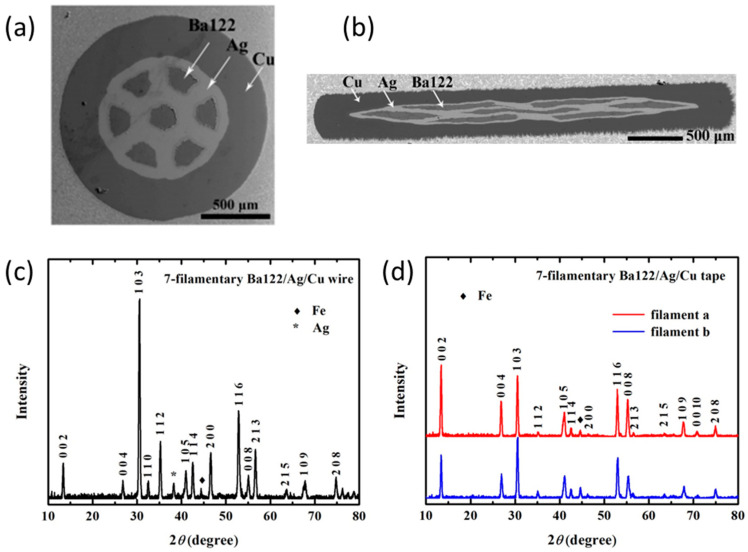
HIP-processed seven-filament (Ba,K)Fe_2_As_2_ superconducting wires and tapes. (**a**) SEM image of a cross section of the wire; (**b**) SEM image of a cross section of the tape; (**c**) XRD pattern of the wire; (**d**) XRD pattern of the tape [61]. Adapted with permission from Ref. [61]. 2014, Elsevier.

Liu et al. fabricated seven-filament (Ba,K)Fe_2_As_2_ superconducting wires and tapes through the HIP process in the same year [61]. As is shown in the XRD patterns of Figure 16, the *c*-axis texture of the tapes is significantly stronger than that of the wires. It is proven that the flat-rolling process in the fabrication of the tapes is the primary producer of *c*-axis texture, while HIP has no significant influence on texture. For the wires, Ba-122 grains in SEM images are well grown, plate-like, but randomly orientated, and the filament is highly dense. All seven filaments have an average Hv above 200, indicating a great improvement in grain connectivity. For the tapes, well-grown plate-like grains were aligned roughly parallel to the tape surface. The filament in the center had relatively lower hardness. XRD patterns also reveal a clear difference in the degree of texture between filaments, indicating deformation variation between filaments in the tape. The transport *J*_c_ of the wires and tapes reached 1.3 × 10^4^ A cm^−2^ (4.2 K, 10 T) and 4.8 × 10^4^ A cm^−2^ (4.2 K, 10 T), respectively. It is the first study to reported *J*_c_ values for HIP-sintered multi-filamentary IBS tapes and wires. It is demonstrated that the HIP process is also suitable for multi-filament IBS wires and tapes, and copper is an ideal sheath metal for IBSs [61]. They also fabricated (Ba,K)Fe_2_As_2_ superconducting tapes using flat-rolling and HIP processes [59]. The transport *J*_c_ of the tapes reached 1.1 × 10^5^ A cm^−2^ (4.2 K, 10 T), which is the highest value among IBS wires and tapes produced using the HIP process.

**Figure 17:**
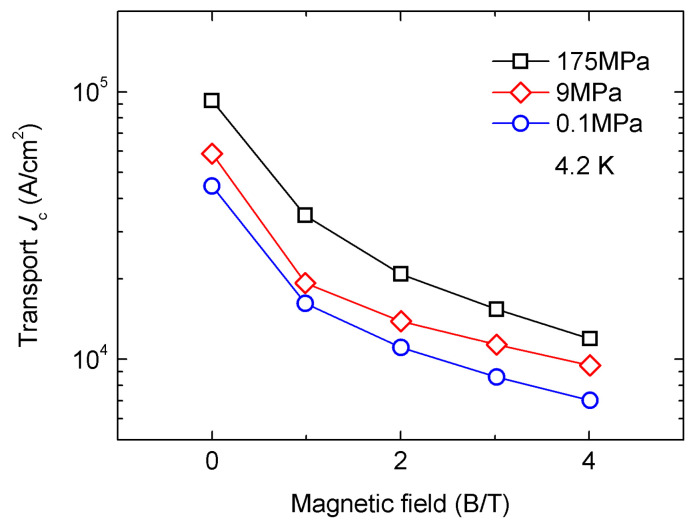
Transport *J*_c_ of the CaKFe_4_As_4_ samples heat-treated at 175 MPa, 9 MPa, and 0.1 MPa. Some data are based on [62].

Other types of IBS wires can also be enhanced by the HIP process. In 2018, Pyon et al. fabricated CaKFe_4_As_4_ wires through the HIP method [62]. Copper and silver composite-sheathed wires were sintered at 700 °C under different pressure and time, i.e., 175 MPa for 4 h, 9 MPa for 0.5 h, and 0.1 MPa for 0.5 h. The transport *J*_c_ of the wire under 175 MPa reached 9.6 × 10^4^ A cm^−2^ (4.2 K, 0 T) and 7.6 × 10^3^ A cm^−2^ (4.2 K, 10 T), higher than those of the HIP wires under lower pressures, as is shown in Figure 17. It is shown that the *J*_c_ and the core density were strongly affected by the pressure of HIP. A decomposition of CaKFe_4_As_4_ was shown in the XRD pattern. The factor restraining *J*_c_ is the partial decomposition of CaKFe_4_As_4_ into KFe_2_As_2_, rather than the reaction between the CaKFe_4_As_4_ filament and the silver sheath.

In 2019, Cheng et al. used the HIP method in the fabrication of CaKFe_4_As_4_ tapes [63]. The CaAs, KAs, Fe, and As powders were mixed with the nominal composition of Ca_1.14_K_1.05_Fe_4_As_4.05_. The Cu/Ag-sheathed tapes were HIP-sintered at a pressure of 150 MPa at 600 °C for 1 h. The transport *J*_c_ observed was 2.1 × 10^5^ A cm^−2^ (4.2 K, 0 T) and 2.2 × 10^4^ A cm^−2^ (4.2 K, 10 T).

The parameters of iron-based wires and tapes fabricated using the HIP process are shown in Table 4 (Parameters of iron-based wires and tapes fabricated by the HIP process [50,52,53,54,55,57,58,59,60,61,62,63,64]). It is shown that the pressure of the HIP process for iron-based wires varies from 120 to 200 MPa, and the temperature is around 700 °C. Temperature was limited because of the low melting point of the Cu/Ag composite sheath (~770 °C) [49], so research into new sheath materials is required to explore the effect of the HIP process at high temperatures. The time required for the HIP process has a negative correlation with the temperature, and tapes require a shorter time than wires. The reason could be that high temperatures and thin sheaths reduce the mechanical strength of the tape, thus making it easier to press. Two shapes of IBS were developed, and each of them has its advantages. Tapes easily obtain a higher degree of axial grain texture, which significantly enhances the transport *J*_c_ of the superconducting filament. Wires are more suitable for applications, including the production of coils. It is notable that the works mentioned are predominantly based on single-filament iron-based wires and tapes, while the HIP process of multi-filament wires and tapes still requires further research.

## 3. Concluding Remarks

**Figure 18:**
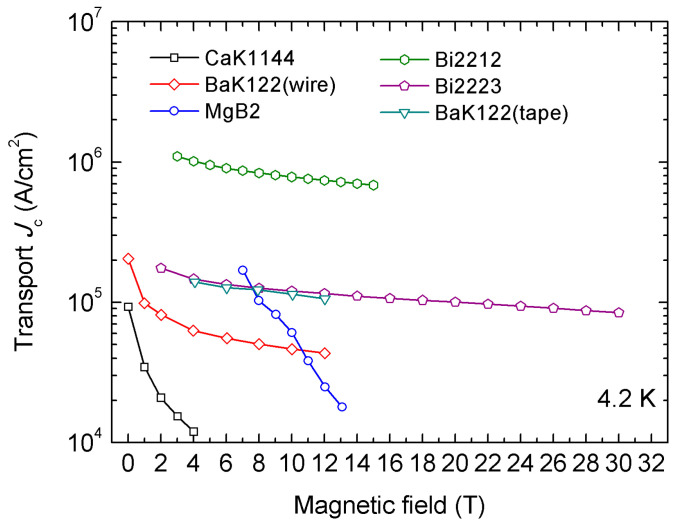
Transport *J*_c_ of overpressured Bi-2212 [29] and Bi-2223 [65] wires and HIP-processed MgB_2_ [42], (Ba,K)Fe_2_As_2_ [58,59], and CaKFe_4_As_4_ [62] wires and tapes.

The development and application of the HIP process in superconducting wires and tapes has been discussed in this paper. The overpressure method has been widely used in the fabrication of Bi-series superconducting wires and tapes, including Bi-2223 and Bi-2212 wires and tapes. Additionally, the HIP process has been used in MgB_2_ and IBS wires and tapes. A general graphic overview of the critical current density of those wires and tapes is shown in Figure 18.

The influence of parameters of the HIP process on the microstructure and properties of superconducting wires and tapes is the focus of current research. Temperature and pressure affect the size of the grain, the formation of heterophase structures, gas thermal expansion, grain contact, element diffusion, and grain recrystallization growth. The optimum HIP process parameters mainly depend on the material of the superconducting filament and will be limited and affected by different sheath materials and the number of superconducting filaments. For example, hard materials such as MgB_2_ require higher pressure than Bi-based wires. The superconducting filament section area of multi-core superconducting wires is smaller, which requires finer grain size; thus, process parameters need to be further refined.

The irreplaceable advantage of the HIP process among heat treatments is that it is suitable for large-scale preparation of superconducting wires and tapes, especially round wires. Wires with a length of over 100 m are key to applications involving superconducting magnets, power transmission, and other fields. Commercial production of km level Bi-2223 multi-core superconducting tapes has been realized using overpressure heat treatment in an HIP furnace. It is predicted that long MgB_2_ and IBS wires and tapes will be improved by the HIP process.

In summary, the HIP process improves the density and grain connectivity of the superconducting filaments of wires and tapes, thus achieving better transport performance than atmospheric-pressure sintering. Further research can be conducted to optimize the parameters of the HIP process for different superconducting wires and tapes. The HIP process would also be suitable for new kinds of superconducting wires and tapes fabricated using the PIT method.

## Figures and Tables

**Table 1:** Parameters of Bi-2223 tapes fabricated using the overpressure process.

Author	Shape	Length	Section Size	*P*_total_ (Mpa)	*p*O_2_ (Mpa)	*T*_max_ (°C)	Time	*I*_c_ (77 K, 0 T) (A)
Rikel (2001) [17]	85-filament tape	3–4 cm	-	17.5	0.012~0.005	815	36 h	100
Kobayashi (2005) [18]	multi filament tape	1500 m	4.5 × 0.24 mm	30	0.02~0.004	-	-	100
Tajima (2013) [22]	121-filament tape	-	4.2 × 0.22 mm	10	0.003	820	24 h	124

**Table 2:** Parameters of Bi-2212 wires fabricated using the overpressure process.

Author	Shape	Length	Section Size	*P*_total_ (MPa)	*p*O_2_ (MPa)	*T*_max_ (°C)	*J*_c_ (kA/cm^2^)	*J*_e_ (kA/cm^2^)
Reeves (1997) [23]	tape	4–8 cm	0.14 mm thick	0.81	0.1	895	270 (4.2 K, 0 T)	-
Larbalestier (2014) [26]	18 × 37 wire	30 m	0.8 mm	10	0.1	-	-	100 (4.2 K, 5 T)
Miao (2014) [28]	85 × 18 wire	1.2 m	1.2 mm	1	0.1	-	-	55 (4.2 K, 15 T)
Jiang (2019) [29]	55 × 18 wire	400 m	0.8 mm	5	0.1	884~894	664 (4.2 K, 15 T)	132 (4.2 K, 15 T)
Shen (2019) [32]	55 × 18 wire	140 m	0.8 mm	5	0.1	-	-	136.5 (4.2 K, 15 T)
Jiang (2021) [31]	85 × 18 wire	9 cm	0.8–1.2 mm	5	0.1	885~890	100 (4.2 K, 5 T)	-

**Table 3:** Parameters of MgB_2_ wires fabricated by the HIP process.

Author	Shape	Section Size	*P* (GPa)	*T* (°C)	Time	*J*_c_ (4.2 K, 0 T) (kA/cm^2^)	*J*_e_ (4.2 K, 0 T) (kA/cm^2^)
Gajda (2015) [40]	18-filament wire	0.83 mm	1	700	15 min	~100	-
Gajda (2016) [41]	wire	0.9 mm	1.1	740	40 min	~40	-
Jie (2017) [42]	wire	0.83 mm	1.4	700	20 min	-	80
Gajda (2018) [43]	18-filament wire	0.79 mm	1.1	570	210 min	-	38

**Table 4:** Parameters of iron-based wires and tapes fabricated using the HIP process.

Author	Type	Shape	Section Size	*P* (GPa)	*T* (°C)	Time	*J*_c_ (4.2 K, 0 T) (kA/cm^2^)	*J*_e_ (4.2 K, 0 T) (kA/cm^2^)
Weiss (2012) [50]	(Ba,K)Fe_2_As_2_	wire	1.35 mm	192	600	10 h	120	10
Pyon (2014) [52]	(Sr,K)Fe_2_As_2_	wire	1.2 mm	120	700	4 h	100	9.4
Pyon (2016) [53]	(Ba,K)Fe_2_As_2_	wire	1.2 mm	175	700	4 h	175	20
Pyon (2016) [53]	(Ba,K)Fe_2_As_2_	tape	0.4 mm thick	175	700	4 h	254	23
Liu (2017) [54]	(Ba,K)Fe_2_As_2_	wire	1.5 mm	200	700	4 h	76	9.4
Pyon (2018) [59]	CaKFe_4_As_4_	wire	1.2 mm	175	700	4 h	96	7.6
Liu (2019) [55]	(Ba,K)Fe_2_As_2_	tape	0.3 mm thick	200	740	1 h	190	58
Miyawaki (2019) [64]	(Ba,Na)Fe_2_As_2_	wire	1.2 mm	175	700	4 h	76	24
Cheng (2019) [63]	CaKFe_4_As_4_	tape	0.4 mm thick	150	600	1 h	210	22
Pyon (2020) [57]	(Ba,Na)Fe_2_As_2_	wire	1.2 mm	175	700	4 h	204	40
Guo (2021) [58]	(Ba,K)Fe_2_As_2_	wire	1.5 mm	150	700	4 h	200	47
Pyon (2021) [60]	(Ba,Na)Fe_2_As_2_	wire	1 mm	200	700	4 h	-	54
Liu (2021) [61]	(Ba,K)Fe_2_As_2_	7-filament wire	1.5 mm	150	740	2 h	-	13
Liu (2021) [61]	(Ba,K)Fe_2_As_2_	7-filament tape	0.3 mm thick	150	740	1 h	-	48
Liu (2021) [59]	(Ba,K)Fe_2_As_2_	tape	0.3 mm thick	150	740	1 h	114 (10 T)	-

## Data Availability

Not applicable.
